# Miscellaneous

**Published:** 1857-11

**Authors:** 


A DETECTOR DETECTED. — Having had occasion of late to expose some of the frauds that are so often perpetrated upon the literature of our profession, we were not a little astonished, some weeks ago, to see ourselves accused of harboring one of the worst of thieves, and such, upon examination, we found to be strictly true.

Some months ago, a medical gentleman of this city handed us for inspection, with a view to publication, a paper entitled "Strabismus, with some Novel Suggestions respecting the Operation for its Relief. By Thomas Graham, M.D., F.R.C.S., &c., late of Sydney, Australia," saying, at the same time, that he had a slight acquaintance with the author, who appeared to be a gentleman, and whose claim to the title attached to his name was substantiated by an exhibition of his diploma from the Royal College of Surgeons. Upon reading the article, we concluded to publish it, and did so in the March number of this journal, when lo and behold it proved to be a verbatim copy from a communication of Mr. Critchett to the pages of the Lancet.

Such is our connection with the article in question, and such the rascality of the said Thomas Graham, F.R.C.S., late of Sydney, Australia, now supposed to be lurking about London. It is not unlikely that we may have seen Mr. Critchett's article in the Lancet, but we defy any one who is compelled to read from fifty to seventy-five periodicals per year to carry with him such an accurate recollection of their contents as to be able at once to detect an imposition such as we have been subjected to. We hope the Lancet or some other British periodical will do us the favor to expose the scoundrel who has thus attempted to steal his way into professional notice.

—

PROFESSOR VON MAUTHNER, OF VIENNA. — This distinguished and learned physician paid a visit to the medical, scientific, and charitable institutions of this city last September, and expressed himself much gratified with what he saw. Dr. Mauthner is the founder of the Children's Hospital at Vienna, one of the most celebrated establishments of the kind in Europe, the author of several able works on infantile diseases, and the editor of a journal expressly devoted to infantile pathology and therapeutics. His treatise on the "Dietetics of Children" is probably the best work that has ever appeared on the subject, and is well deserving of a translation into the English language.

Philadelphia was also favored, during the past summer, with a visit from Dr. Voss, Professor of Anatomy in Christiana University, Sweden. We had the pleasure of several interviews with him, and were most favorably impressed with his great intelligence and remarkable modesty. The visits of such men as Professors Voss and Mauthner cannot fail to exert a favorable influence upon their learned countrymen in regard to the medical men and medical institutions of the United States.

—

PATHOLOGICAL SOCIETY OF PHILADELPHIA.—Under this name a society has just been organized in this city, consisting principally of young men, who already occupy prominent positions in the profession, or who are rapidly rising to distinction and usefulness. The meetings are to be held once a fortnight throughout the year, and the exercises are to consist in the exhibition of specimens in Pathological Anatomy, and in the reading of papers upon kindred topics. Judging from the character and intelligence of the gentlemen composing the association, there is reason to believe that the proceedings will be conducted with great spirit, and that they will be productive of much good to the profession of Philadelphia, as well as the profession at large.

The officers of the society for the present year are,—

Dr. S. D. Gross, President.

Dr. R. La Roche, Dr. Alfred Stillé, Vice-Presidents.

Dr. J. Da Costa, Secretary.

Dr. Thos. G. Morton, Assistant-Secretary.

Dr. A. Hewson, Treasurer.

—

MORALS OF THE VIENNESE.—From the "Times and Gazette" we learn that, by the statistics published under the auspices of the Common Council Bureau of Vienna, the number of illegitimate births in that city has almost equalled the number of the legitimate births during the four years from 1853 to 1856. The following are the figures on the subject: 1853. Legitimate births,

11,264; illegitimate, 10,686. 1854. Legitimate births, 11,252; illegitimate, 10,801. 1855. Legitimate births, 10, 650; illegitimate, 9,522. 1856. Legitimate, 10,870; illegitimate, 10,311.

—

LAUREL HILL CEMETERY.—During a recent visit to this beautiful city of the dead, we had an opportunity of seeing the monument which has been erected by the "Philadelphia Contributors" in memory of the physicians, druggists, and nurses, of this city, who volunteered to aid the sufferers by yellow fever at Norfolk and Portsmouth, Virginia, in 1855, and died in the discharge of their duties,—*martyrs to the cause of humanity.* It is a beautiful column of white marble, about twenty-five feet high, with appropriate devices and inscriptions, and comprises the names of Drs. Thomas Craycroft, Courtlen Cole, and Herman Kierson, with that of Edmund Barrett, Student of Medicine.

—

BUFFALO MEDICAL JOURNAL.—This journal is now edited by Professor Hunt and Dr. Austin Flint, Jr., the worthy son of its worthy founder. Dr. Flint is already favorably known by several valuable articles which he has contributed to periodical medical literature, and we greet his advent to the editorial corps with no ordinary satisfaction, knowing that his pen will do good service in the cause of American journalism.

—

MEDICAL INDEPENDENT.—We are gratified at the improved appearance of this journal, and welcome the addition of Professor Gunn to the editorial chair. In the October No. is an able and manly advocacy of such a clinical course in the University of Michigan, as the wants of medical students of the present day imperatively demand. We sincerely wish the Independent success in its endeavors to stimulate the Regents to place the Medical Department of the University upon a true hospital basis.

—

WE are gratified to learn that Messrs. Lippincott & Co. are about to put to press an English translation of Mons. Malgaigne's celebrated treatise on Fractures and Dislocations, by John H. Packard, M.D., of this city. The work will be accompanied by valuable additions by the American editor, and will be illustrated by numerous woodcuts, executed in the best style of the art. It need hardly be added that such a work has long been needed by the profession of this country.

—

ENLARGEMENT AND IMPROVEMENT OF THE REVIEW.—Beginning with the next year, the Publishers, with their well-known enterprise and liberality, have concluded to add to each number of this journal thirty-two pages, to be devoted principally to an analysis of the progress of American Medicine and Surgery for the preceding year. The volume, when thus enlarged, will contain a greater number of pages than any medical periodical published in this country, and the small addition to the subscription price will, we doubt not, be cheerfully submitted to by the present subscribers.

The enlargement, as just intimated, is intended to provide for a new and important improvement, one which has never been attempted in this country, so far as we know, and we are not aware that any of the European journals pursue the plan which we have adopted. It must be well known to every one, that under the present system of making extracts from the various journals, the most valuable papers, on account of their length, oftentimes pass almost entirely unnoticed, while many of little or no merit are universally copied on account of their brevity. To classify and to analyze these papers, and thus to exhibit what we, on this side of the Atlantic, are doing to advance the profession, is the object of the present enterprise. For this purpose, we have engaged the services of some of the most able men in the country, and have allotted to each his particular sphere of duty. The division of subjects which we have adopted is as follows:

1. Anatomy and Physiology.
2. Surgery.
3. Medicine.
4. Materia Medica and Therapeutics.
5. Obstetric Medicine.
6. Chemistry, Toxicology, and Medical Jurisprudence.

The work is already in progress, and the first number of the coming volume will contain a carefully elaborated digest of the discoveries and improvements made in anatomical and physiological knowledge in the United States during the past ten years. The other subjects will appear in the order above-mentioned in the successive issues of the journal throughout the entire year. The names of the individuals by whom the articles are prepared will always be appended.

In addition, it is our intention to furnish from time to time analyses of the more valuable papers that have appeared in the older medical journals now extinct, and, therefore, not accessible to the members of the profession generally; and we hope thereby to enlighten many of our readers in regard to what our forefathers accomplished for scientific medicine, and to revive a knowledge of those who have honored the profession, and distinguished themselves, but whose names and works are now known only to a few.

